# Activated hydrochar from areca sheath biomass for CO_2_ capture: adsorption performance and life cycle challenges

**DOI:** 10.1039/d6ra08488e

**Published:** 2026-11-05

**Authors:** Sooraj Mohan, Ranjan Kumar Ghadai, Jana Petrů, Sushant S. Satputaley, P. Dinesha

**Affiliations:** a Manipal Institute of Technology, Manipal Academy of Higher Education Manipal India dinesha.p@manipal.edu; b Faculty of Mechanical Engineering, VŠB – Technical University of Ostrava 70800 Czech Republic; c Department of Mechanical Engineering, St. Vincent Pallotti College of Engineering & Technology Nagpur Maharashtra India

## Abstract

The development of sustainable CO_2_ capture technologies is critical in the present global drive to combat climate change. Hydrochar, obtained from hydrothermal carbonization (HTC), has demonstrated capabilities to adsorb CO_2_. This study evaluates activated hydrochar derived from areca sheath biomass for CO_2_ capture, associated with a life cycle assessment (LCA) study. Hydrochars were produced at 180 °C (AS-180), 200 °C (AS-200), and 220 °C (AS-220), followed by chemical activation using KOH at a KOH : hydrochar mass ratio of 3 : 1 and carbonization at 700 °C for 2 h to enhance microporosity. AS-200 achieved the highest surface area (144 m^2^ g^−1^), outperforming AS-180 and AS-220. Life cycle assessment of sorbent production reveals pronounced differences in climate change impacts, with AS-200 exhibiting the lowest carbon footprint (235 kg CO_2_-eq. per kg), followed by AS-220 (611 kg CO_2_-eq. per kg) and AS-180 (941 kg CO_2_-eq. per kg). Across all cases, chemical activation using KOH dominates emissions (about 67%), while fossil-derived thermal energy contributes about 16%, identifying upstream chemical manufacture and energy supply as primary hotspots. AS-200 achieves the highest measured CO_2_ adsorption capacity (2.5 mmol g^−1^). Under the baseline inventory with an assumed 1% sorbent makeup, its adsorption–regeneration LCA gives 2.55 kg CO_2_-eq. per kg CO_2_ processed, with fresh-sorbent production contributing substantially. This modelled climate burden is conditional on the replacement assumption; cyclic stability and the actual replacement requirement were not established. The baseline assessment identifies upstream sorbent manufacture as a priority for reducing emissions. Wastewater-treatment burdens are excluded from these baseline totals.

## Abstract 2

The development of sustainable CO_2_ capture technologies is critical in the present global drive to combat climate change.

## Graphical abstract

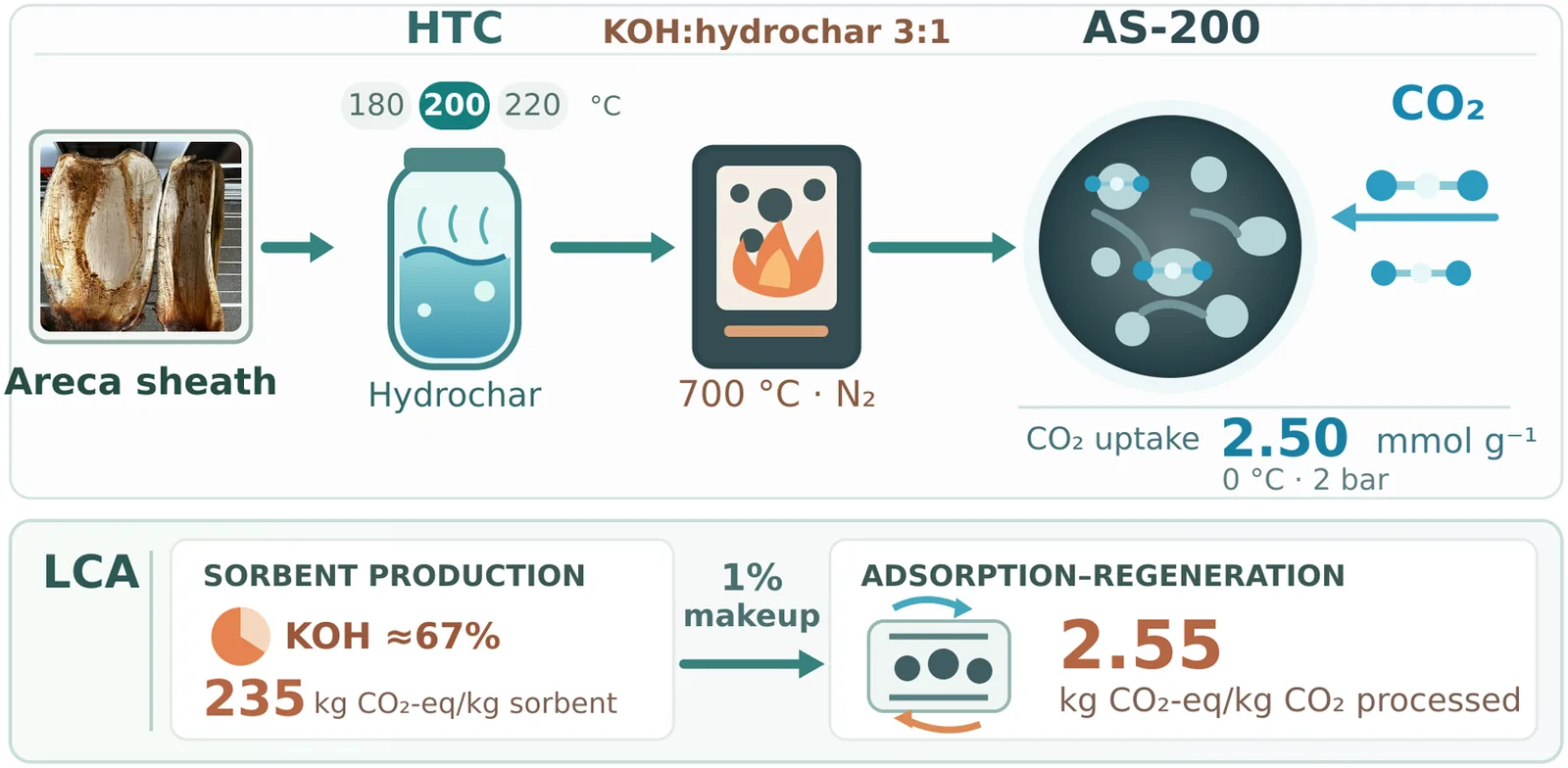

## Introduction

Greenhouse gases (GHGs) such as carbon dioxide (CO_2_), methane (CH_4_), and nitrous oxide (N_2_O) play a crucial role in regulating the Earth's temperature by absorbing and re-emitting infrared radiation in the atmosphere. This natural process forms the basis of the greenhouse effect, which is essential for maintaining temperatures suitable for life. Among these gases, carbon dioxide is the most abundant and therefore has the greatest impact on global warming compared to methane and nitrous oxide.^1,2^ While all three are necessary in small amounts, their concentrations have increased significantly due to human activities like fossil fuel combustion, industrial processes, and large-scale agriculture, leading to intensified global warming and climate change.^3^

Since the 1970s, emissions of CO_2_, CH_4_, and N_2_O have risen steadily, with CO_2_ alone accounting for approximately 73.5% of global GHG emissions.^1^ As part of mitigation efforts, carbon capture and storage have gained widespread attention. This technique involves capturing CO_2_ from large point sources and storing it in geological formations. Among the different carbon capture methods, adsorption stands out as a cost-effective and energy-efficient solution.^4^ Adsorption systems can offer high CO_2_ capacity, low energy requirements for regeneration, fast reaction kinetics, and minimal pressure drops, making them highly attractive for large-scale use.^5^ As demand for effective adsorbents increases, carbon-based materials such as activated carbon, biochar, and hydrochar have emerged as promising options. These materials are valued for their high surface area, tunable pore structures, ease of regeneration, and low production costs.^6,7^ Water affinity varies with feedstock, thermal treatment, and surface chemistry; biochars may be hydrophobic or hydrophilic. Marshall *et al.* found hydrophobicity in grapevine biochars produced at 400 °C but not at higher pyrolysis temperatures.^8^ In humid gas, water can bind to polar groups and form clusters that restrict CO_2_-accessible pores.^9^ Oxygen-containing groups can therefore contribute to both CO_2_ interactions and water affinity, making humidity tolerance material- and condition-dependent. Recent reviews have also highlighted that surface area, microporous structure, and oxygen-containing functional groups are the primary factors governing CO_2_ adsorption in engineered biochars, while chemical activation remains one of the most effective approaches for enhancing adsorption performance.^10^

Biochar and hydrochar are typically produced *via* thermochemical methods such as pyrolysis and hydrothermal carbonization (HTC). However, when dealing with high-moisture biomass, HTC is generally preferred because it eliminates the need for expensive pre-drying steps required by pyrolysis. The HTC process enables the efficient conversion of biomass into a carbon-rich product, hydrochar, under elevated temperatures and pressures in an aqueous environment.^11^ This method captures most of the carbon content in the feedstock and is less dependent on moisture levels, making it highly energy efficient. Reaction conditions, such as temperature (usually between 180–250 °C) and time, determine the extent of biomass decomposition. Pyrolysis remains one of the most widely employed thermochemical routes for producing activated carbons, particularly when combined with steam or chemical activation to generate highly porous structures. However, pyrolysis generally requires drying of high-moisture biomass prior to carbonization,^12^ which may increase process energy demand depending on the feedstock characteristics. Consequently, the relative environmental advantages of HTC- and pyrolysis-derived activated carbons depend on both the biomass properties and the downstream activation process.

Goel *et al.*^13^ prepared the KOH-activated hydrochar from banana peel using HTC at 180 °C, 200 °C, and 220 °C. The study reported that higher temperature HTC showed lower porosity, and maximum CO_2_ capture was obtained for the hydrochar prepared at 180 °C. Deepak *et al.*^14^ studied the process temperature of HTC on the porosity and CO_2_ capture ability of hydrochar derived from orange peel. The orange peel produced at 220 °C exhibited the maximum CO_2_ capture of 3.045 mmol g^−1^. Lilian *et al.*^15^ used sawdust to prepare KOH-activated hydrochar and investigated CO_2_ capture. The HTC was carried out at a temperature range of 150–250 °C for a 1 h duration. The chemical activation was carried out by treating with KOH and heating at 650–850 °C for 90 min. The CO_2_ capture was performed using TGA at 40 °C and 75 °C. The activated hydrochar showed better surface area and pore volume, which in turn resulted in higher CO_2_ capture. Similarly, studies related to hydrochar derived from rice husk,^16^ pomelo peel,^17^ jackfruit peel^18^ showed better porosity and higher CO_2_ capture capacities.

Biochar-based CO_2_ sorbents have been developed by modifying both pore structure and surface chemistry. Kwon *et al.* prepared KOH-treated bamboo and orange-peel biochars by pyrolysis at 500 °C. Their reported uptakes at 273 K and 1 bar were 0.63 and 1.01 mmol g^−1^, respectively; the orange-peel material also showed a greater abundance of oxygen- and nitrogen-containing functionalities.^19^ This comparison illustrates the influence of feedstock composition under common preparation conditions. Sreńscek-Nazzal *et al.* investigated banana-peel biochar activated with KOH, with and without urea modification. Urea increased nitrogen incorporation and CO_2_ uptake for most of the studied preparation conditions, while activation developed microporous and mesoporous structures.^20^ These modifications can change adsorption-site chemistry and accessible pore volume together, so the uptake enhancement cannot be attributed to nitrogen content alone.

Wu *et al.* co-carbonized bamboo shoot shells with urea and subsequently activated the precursor with K_2_CO_3_. Their BNC-800-3 adsorbent reached 7.52 mmol g^−1^ at 0 °C and 3.60 mmol g^−1^ at 25 °C, both at 1 bar, demonstrating the potential of combining nitrogen incorporation with narrow-pore development.^21^ These established approaches show that adsorption performance depends on the feedstock, activation conditions, and surface modification. Uptake alone does not account for the chemicals and energy required to prepare and regenerate a sorbent. More intensive modification can improve capacity while increasing activator and energy inputs; their climate burdens must therefore be considered alongside adsorption performance. This motivates the linked material and LCA assessment undertaken here.

Wahby *et al.* reviewed biochar-based engineered materials for CO_2_ removal from laboratory studies towards industrial applications, emphasizing that improved uptake must be accompanied by selective adsorption, repeatable regeneration, practical sorbent shaping, and viable preparation costs.^22^ This process perspective complements material comparisons by connecting pore and surface modification with the operating requirements of adsorption beds.

The contribution of this study is a combined assessment of areca sheath hydrochar that links HTC precursor conditions, measured CO_2_ adsorption, and the modelled climate burden of sorbent manufacture and use. HTC followed by KOH activation is an established preparation route. Earlier studies reported the highest uptake at 180 °C HTC for banana peel hydrochar and at 220 °C HTC for orange peel hydrochar.^11,12^ These contrasting responses motivate a feedstock-specific comparison of HTC conditions. Here, areca sheath hydrochars prepared at 180, 200, and 220 °C are compared using a common KOH : hydrochar mass ratio of 3 : 1 and activation at 700 °C for 2 h. This design evaluates the response of different HTC precursors to the same activation treatment. The measured adsorption performance is considered alongside yield, morphology, surface chemistry, and local elemental composition. The assessment then adds two environmental reporting bases: production of 1 kg of activated hydrochar and processing of 1 kg of CO_2_ in the baseline adsorption–regeneration inventory. Linking these evaluations identifies chemical and energy burdens that BET area or uptake alone cannot represent. The chemical question is how precursor HTC conditions, followed by the same KOH activation and washing, relate to the final oxygen-containing environments, retained potassium, and CO_2_ uptake. The study therefore compares these relationships for areca sheath and examines whether favourable adsorption performance also corresponds to a lower modelled climate burden. The use-stage assessment remains conditional on the assumed 1% makeup and does not establish cyclic service life. EDX supports this comparison by examining potassium retained after washing; detecting the constituent elements is not presented as a separate methodological novelty. Studies combining biomass-derived CO_2_ sorbents with LCA have already been reported. Yuan *et al.* assessed pyrolyzed, KOH-activated food-waste biochar,^23^ while Monteagudo *et al.* examined gasification-derived olive-pomace biochar for direct air capture.^24^ The present study addresses a different feedstock and preparation route: it compares three areca-sheath HTC temperatures under identical subsequent activation conditions and evaluates both sorbent-production and CO_2_-processing climate burdens.

## Materials and methods

### Synthesis of hydrochar

The areca (*Areca catechu* L.) sheath was collected from a local plantation. The semi-dried areca sheath is shown in Fig. 1. The feedstock drying was carried out at 90 °C till the weight became constant. Then, powdering was performed to reduce the particle size to 20 µm for efficient hydrothermal carbonization. Fig. 2 shows the steps involved in the synthesis of activated hydrochar. The HTC temperature was controlled using an autotransformer by adjusting the power input. 4 g of dried fine powered feedstock was mixed with 60 g of water and placed inside the HTC reactor. The heating was carried out at 180 °C, 200 °C, and 220 °C for 3 h. After cooling the reactor, the hydrochar was filtered and dried to remove the moisture. Chemical activation was conducted by impregnating the hydrochar with a 6 M KOH solution for 6 h, using a KOH : hydrochar mass ratio of 3 : 1, based on the dried hydrochar obtained after HTC (3 g of KOH per 1 g of dried hydrochar). The impregnated hydrochar was dried and then heated under nitrogen flow at a rate of 5 °C min^−1^ to 700 °C, where it was maintained for 2 h for thermal activation/carbonization. After thermal activation, the samples were washed with hydrochloric acid and deionized water, followed by a final drying step of 6 h. The prepared activated hydrochar was named AS-180, AS-200, and AS-220, respectively, for the three temperatures 180 °C, 200 °C, and 220 °C.

**Fig. 1:**
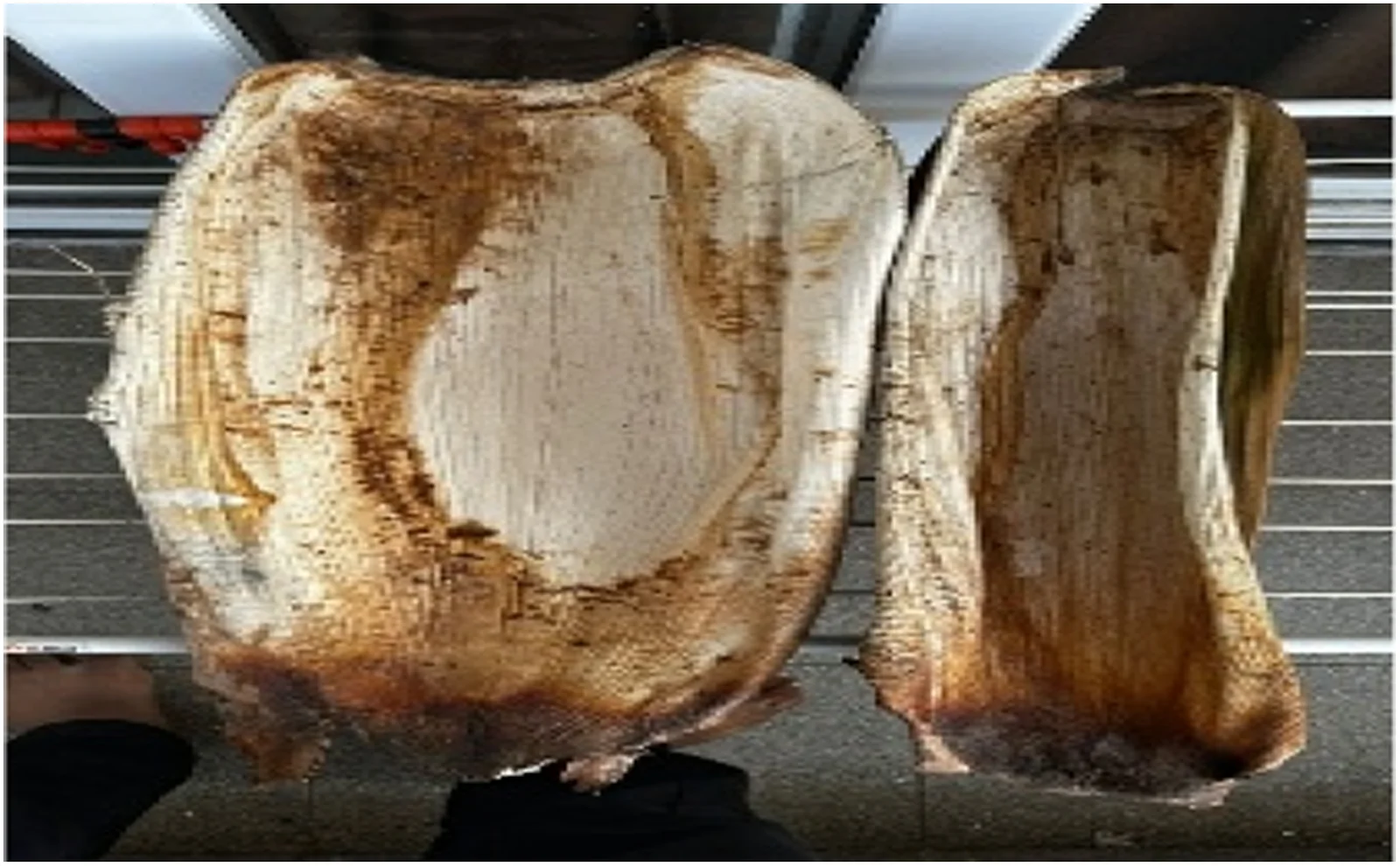
Semi-dried areca sheath used in this study.

**Fig. 2:**
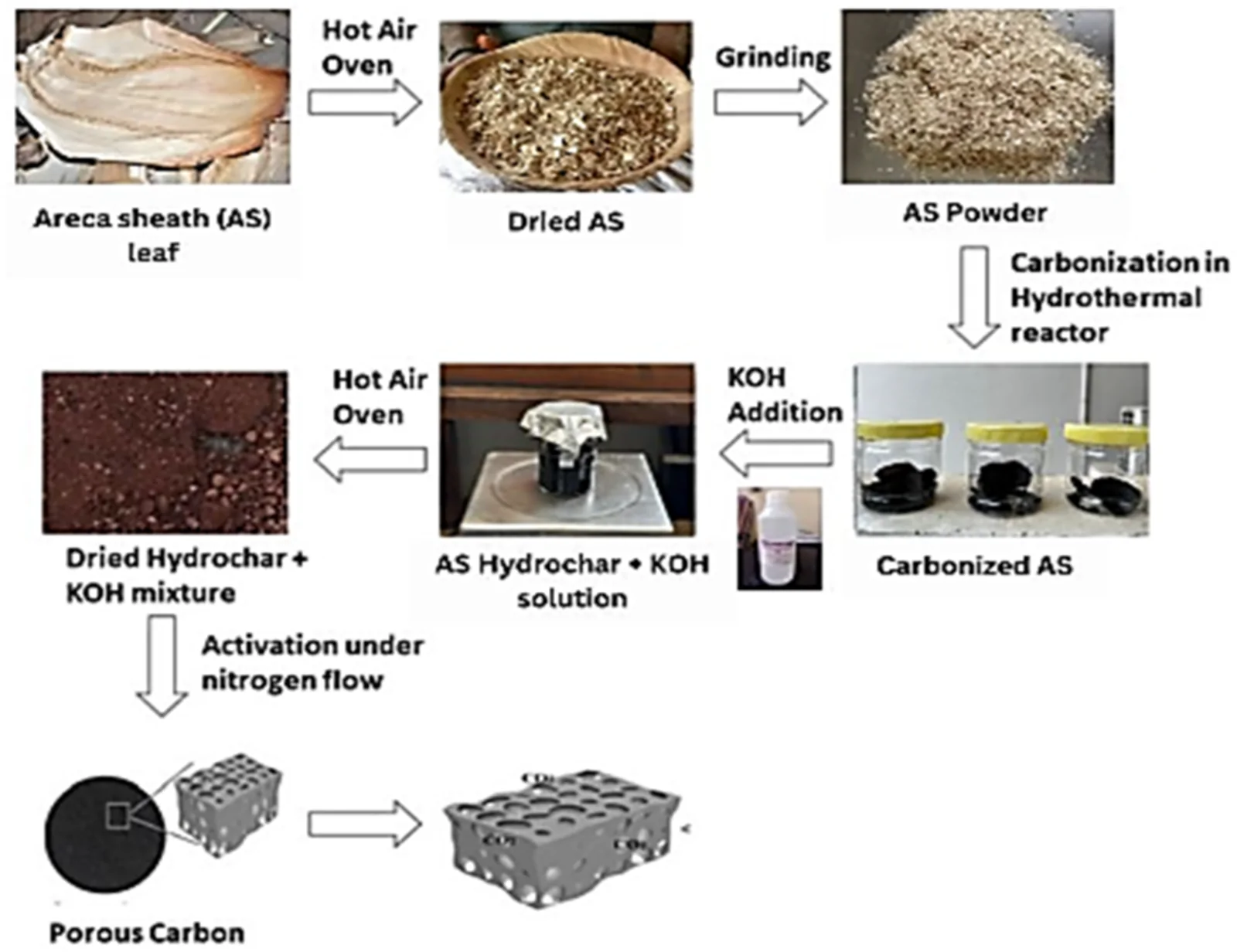
Stages of activated char preparation.

### Hydrochar characterization

The physicochemical characterization of activated hydrochar was conducted to check the usability of the samples for CO_2_ capture. XRD was used to examine diffraction features of the activated hydrochars, including reflections potentially arising from retained crystalline inorganic residues. The microstructure and surface topography were analyzed using SEM. Textural characterization was performed using a Micromeritics BET (Brunauer–Emmett–Teller) analyzer to determine the nitrogen-accessible specific surface area. The specific surface area was determined by N_2_ adsorption–desorption using a BET surface area analyzer. Prior to analysis, the samples were degassed at 250 °C for 10 h under vacuum to remove moisture and physically adsorbed species. Nitrogen adsorption measurements were carried out at 77 K, and the BET surface area was calculated using adsorption data within the relative pressure (*P*/*P*_0_) range of 0.05–0.30, following the BET method. FTIR was used for functional group analysis to identify the chemical functionalities on the hydrochar surface responsible for gas adsorption. EDX was used to examine the local elemental composition of the prepared activated hydrochars after the washing and drying steps. The reported weight percentages are interpreted as local, semi-quantitative estimates for the quantified elements, rather than as bulk elemental compositions.

### CO_2_ capture

CO_2_ adsorption was measured using an Anton Paar iSorb HP-100 high-pressure volumetric (manometric) gas sorption analyser. The adsorbent mass ranged from 0.020 to 0.055 g per measurement, and CO_2_ with a purity of >99.99% was used. Samples were degassed before analysis. Measurements were performed at 0 °C and 25 °C, with three repetitions for each sample and condition (*n* = 3), at target absolute pressures of 1 and 2 bar. In this volumetric method, adsorption is quantified from the gas introduced into the system and the measured gas pressure. Gas dosing provides the pressure points for the isotherm; a continuous flow rate is not a measurement parameter for this method.

### Life cycle assessment

LCA was conducted to quantify the climate change impacts associated with the production and use of activated hydrochar for CO_2_ capture. Two functional units were considered: (a) 1 kg of activated hydrochar for evaluating sorbent production and (b) processing of 1 kg of CO_2_ for assessing the adsorption–regeneration stage. The system boundary for sorbent production included hydrothermal carbonization, chemical activation with KOH, high-temperature carbonization, washing and drying, and all upstream processes related to energy and material inputs, including electricity supply, chlor-alkali electrolysis for KOH production, hydrochloric acid manufacture, and industrial gas supply. The system boundary includes the upstream production of chemicals, water, electricity, and thermal energy associated with sorbent synthesis. Wastewater treatment was excluded from the baseline inventory because washing-effluent volume and composition were not available. A separate, normalized screening estimate is presented in the LCA discussion and Table 5 using published brine-concentration energy benchmarks; it is not an updated process-specific LCA. For the adsorption–regeneration stage, energy consumption and fresh-sorbent replacement were included using an assumed makeup fraction of 1% in the baseline inventory. This fraction is a modelling input; cyclic capacity retention, physical attrition, and regeneration efficiency were not measured for the prepared hydrochars. Systems-analysis frameworks include sorbent replacement and material properties when assessing capture processes.^25,26^ These frameworks provide context for including replacement, rather than validation of the 1% value for areca sheath hydrochar. The adsorption–regeneration results are therefore reported as conditional on this inventory assumption. Life cycle inventory modelling and impact assessment were performed using the openLCA 2.2 software platform, with background process data sourced from the ecoinvent 3.10 database. Climate change impacts were evaluated using the IPCC 2021 characterization method, and results are primarily reported as Global Warming Potential over a 100 year time horizon (GWP_100_). The original Sankey outputs were used to trace upstream pathways contributing more than 5% of total GWP_100_. Fig. 7 presents conventional bar charts of the baseline totals and an analytical illustration of the AS-200 KOH-linked makeup contribution at 0.5%, 1%, and 2% makeup. These illustrative fractions are not measured replacement rates. The illustration rescales the existing contribution with all other inventory conditions fixed; it is not a rerun of the complete LCA model. No statistical uncertainty intervals are assigned.

## Results and discussion

The physicochemical characteristics of hydrochar samples were compared and analysed. The CO_2_ adsorption was performed with three samples and compared.

### Yield

Three hydrochar samples prepared had yield percentages of 51.5%, 45.62%, and 44.14%, respectively. There was a clear decrease in yield with an increase in reaction temperature. This is expected with the thermochemical decomposition reactions involved in HTC, where a temperature rise facilitates greater decomposition of the volatile organic compounds in the biomass. At higher temperatures, more lignocellulosic material is hydrolyzed, decarboxylated, and dehydrated, resulting in increased liquid and gaseous products and a lower solid hydrochar yield. The decreased yield with higher temperatures can therefore be attributed to greater breakdown of the structural materials of the areca sheath, such as hemicellulose and cellulose, which are thermally more labile than lignin. The reported yields correspond to the hydrothermal carbonization stage. During the subsequent KOH activation and carbonization process, additional mass loss occurred due to the removal of volatile matter and carbon gasification associated with pore development. The burn-off values were 43%, 47%, and 39% for AS-180, AS-200, and AS-220, respectively. The higher burn-off of AS-200 is consistent with its greater nitrogen-accessible surface area after activation. However, burn-off measures overall mass loss and does not identify which pore sizes were generated. The lower burn-off of AS-220 may reflect differences in precursor reactivity after HTC; it does not, by itself, demonstrate micropore collapse or establish a pore-size distribution.

### Energy dispersive X-ray (EDX) analysis

The EDX results refer to the washed, activated hydrochars AS-180, AS-200, and AS-220. They describe local electron-excited X-ray interaction volumes extending beneath the specimen surface; they are neither whole-batch bulk assays nor measurements restricted to the outermost surface layer. Roughness, local heterogeneity, and X-ray absorption can influence quantification.^27^ The reported elemental percentages sum to approximately 100% for each sample and are therefore interpreted as normalized estimates for the quantified elements. For AS-180, the values were C 21.98 wt%, O 38.31 wt%, K 38.27 wt%, Al 0.41 wt%, and Si 1.04 wt%. Fig. 3 compares the local carbon, oxygen, and potassium estimates. The high K fraction identifies a potassium-rich analysed region after washing; it does not establish 38.27 wt% K throughout the sorbent. Given the KOH : hydrochar mass ratio of 3 : 1, activator-derived residue is a plausible major source, although native feedstock potassium cannot be separated from that contribution using these data.

**Fig. 3:**
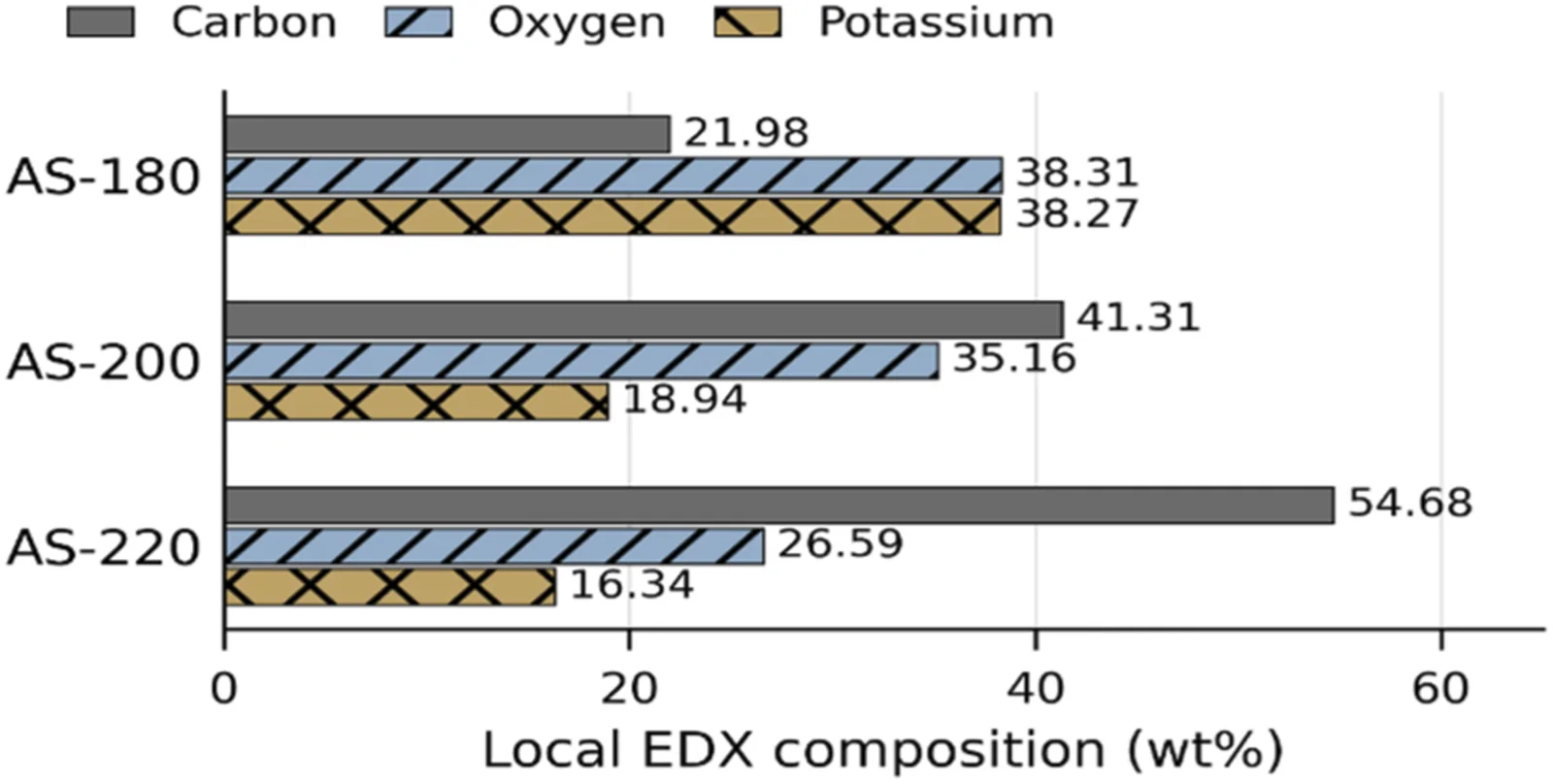
Local EDX estimates for C, O and K after washing (wt%).

For AS-200, the local EDX estimates were C 41.31 wt%, O 35.16 wt%, K 18.94 wt%, Mg 0.43 wt%, Si 3.40 wt%, and Al 0.76 wt%. The lower relative K fraction and higher relative C fraction compared with AS-180 may reflect differences in precursor structure, retention of inorganic material, washing accessibility, and the regions sampled. Because these are normalized compositions of separately prepared samples, a reduction in the relative contribution of K-containing material can increase the reported C percentage without establishing an increase in absolute carbon mass. These measurements consequently do not demonstrate potassium volatilization or quantify potassium removal during washing.

For AS-220, the local EDX estimates were C 54.68 wt%, O 26.59 wt%, K 16.34 wt%, Al 0.37 wt%, and Si 2.02 wt%. Magnesium was not reported for this sample; this does not establish its complete volatilization. The increasing local C fraction and decreasing O and K fractions describe a compositional trend among the analysed regions. EDX alone does not identify oxygen functional groups, graphitic ordering, hydrophobicity, or calorific value. In particular, 16.34 wt% K remains a substantial local fraction, so AS-220 should not be described as a high-purity carbon on the basis of these measurements.

The washing chemistry provides a basis for interpreting potassium retention. HCl neutralizes accessible alkaline potassium species, for example KOH + HCl → KCl + H_2_O; carbonate species can also react with acid and release CO_2_. Water rinsing can then extract dissolved potassium salts. However, dissolution and removal require access of the wash liquid to the residue and transport out of the particles. Enclosed deposits, restricted pore entrances, or agglomerates can impede this process. Etzi *et al.* reported residual K-containing compounds after prolonged HCl washing of KOH-activated carbons, showing that acid treatment does not invariably remove every retained potassium species.^28^ This literature supports a possible retention mechanism, not proof of the location or chemical form of K in the present samples. The post-washing EDX detection is direct evidence of K remaining in the sampled regions. Without a paired potassium mass balance, the fraction removed and washing efficiency cannot be calculated. EDX also cannot distinguish residual salts from carbon-associated potassium species; consequently, neither complete removal nor a specific retained K phase is claimed from EDX alone.

Here, EDX supports the comparison of areca-sheath hydrochars prepared at different HTC temperatures under common activation conditions; detection of C, O, and mineral elements is not itself the novelty. Its specific contribution is evidence of potassium remaining in the washed products. At 25 °C and 1 bar, AS-200 has higher CO_2_ uptake than AS-220 (1.84 *versus* 0.39 mmol g^−1^) despite a higher local K fraction (18.94 *versus* 16.34 wt%); AS-220 also has the highest local C percentage (54.68 wt%). Thus, elemental percentages alone do not explain the performance ranking. EDX constrains interpretation of BET, FTIR, and XRD results by identifying potassium-containing material in the analysed regions, without measuring its individual contribution to adsorption.

### Fourier transform infrared spectroscopy (FTIR) analysis

Fig. 4 shows the FTIR spectra of washed, KOH-activated hydrochars; AS-180, AS-200, and AS-220 denote precursor HTC temperatures, while all samples underwent activation at 700 °C. Labels A–F identify approximate absorption regions, with assignments and qualifications given in Table 4. Absorption appears as a decrease in percent transmittance. Region A (3100–3500 cm^−1^) is consistent with hydrogen-bonded O–H stretching from hydroxyl-containing groups and/or adsorbed water.^29,30^ These features do not establish the survival of intact cellulose or hemicellulose after activation.

**Fig. 4:**
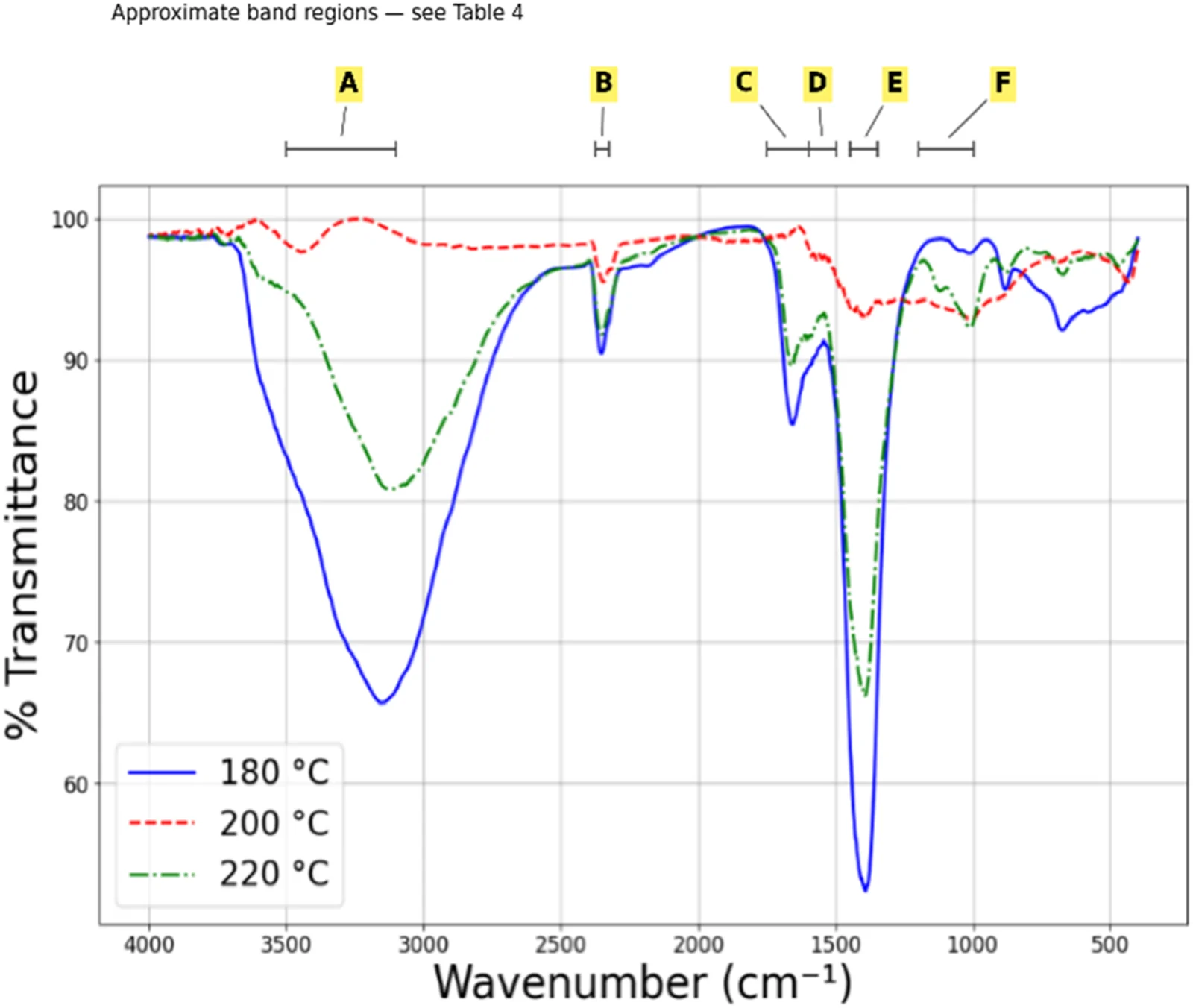
FTIR spectra of AS-180, AS-200, and AS-220. Labels A–F mark approximate band regions assigned in Table 4. Sample labels denote precursor HTC temperatures; all samples were activated at 700 °C.

The 1600–1750 cm^−1^ envelope (C) can contain C=O stretching, including conjugated carbonyl environments. Near 1640 cm^−1^, adsorbed-water bending may overlap this signal. Region D (1500–1600 cm^−1^) is compatible with aromatic skeletal C=C vibrations, with possible conjugated C=O contributions.^34^ Overlap prevents unique identification of individual ketone, aldehyde, or carboxylic-acid species. Aromatic-band intensity alone does not establish an optimum aromatic structure or graphitic ordering.

The conspicuous minimum near 1400 cm^−1^ lies in region E (1350–1450 cm^−1^). C–H deformation can contribute here, while carbonate absorption is another plausible contribution.^29,31^ Carbonate bands near 1420 cm^−1^ have been reported for KOH-treated biochars.^31^ Retained potassium detected by EDX makes an inorganic contribution plausible in the present samples; however, potassium detection and this overlapping band do not identify a specific carbonate phase. The feature is therefore assigned tentatively rather than exclusively to an organic group.

The feature around 2350 cm^−1^ (B) is consistent with CO_2_ asymmetric stretching, whose gas-phase fundamental is approximately 2349 cm^−1^.^32^ A residual atmospheric/background contribution is plausible, and this feature is not used as evidence of C≡C bonding, carbon-network development, or chemically bound CO_2_. Region F (1000–1200 cm^−1^) is tentatively assigned to overlapping C–O/C–O–C vibrations in oxygen-containing environments such as alcohols, phenols, and ethers.^29^

Together, these bands are consistent with oxygen-containing groups and aromatic environments in the activated materials, alongside possible inorganic contributions. The O–H-region depression is more pronounced for AS-180 than AS-220 and is weak for AS-200; the pattern is not a monotonic decline with precursor HTC temperature. Band depth can also depend on sample loading, optical coupling, and background treatment, so the displayed spectra support qualitative assignments rather than quantitative group concentrations or measured dehydration/decarboxylation extents.^29,30^

### Scanning electron microscopy (SEM) analysis

SEM characterization of areca sheath hydrochar samples treated at 180 °C, 200 °C, and 220 °C reveals structural changes related to HTC. The SEM images of the three samples at 20k X magnification are shown in Fig. 5 in the order of temperatures AS-180, AS-200, and AS-220. Raw areca sheath is a densely packed, non-porous structure with rigid fibrillar features typical of lignocellulosic biomass. After HTC, the 180 °C sample shows initial pore development, with partial breakdown of the cellulose–lignin matrix, but limited surface porosity. The 200 °C sample demonstrates the most significant structural reorganization, with irregular cracking, visible voids and pores, and fragmented fibrils-indicative of hemicellulose degradation and lignin softening. At 220 °C, structural collapse occurs, with welded particles and reduced porosity, due to lignin recondensation and thermal repolymerization. SEM observations demonstrating pore development after thermochemical conversion are consistent with previous studies on biomass-derived biochar.^33^ The SEM images describe surface morphology and larger visible pores; they do not resolve sub-nanometre pores or quantify micropore volume.

**Fig. 5:**
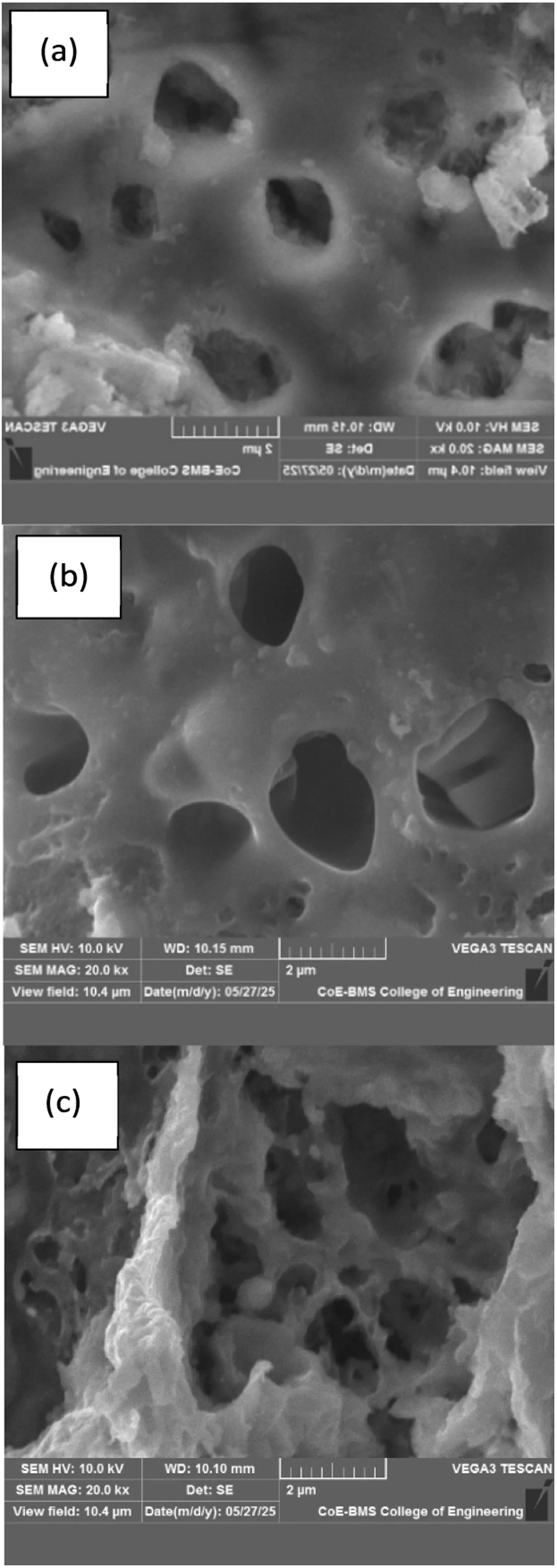
SEM images (at 20k X) for AS-derived carbons of (a) AS-180, (b) AS-200, (c) AS-220.

The nitrogen BET surface areas were 37.3, 144.77, and 135.2 m^2^ g^−1^ for AS-180, AS-200, and AS-220, respectively. These values are relatively low compared with several activated carbons reported in the literature. Although the KOH loading and thermal activation conditions were common to the three samples, HTC changes the composition and degree of condensation of the hydrochar precursor. Consequently, the same activation treatment need not produce the same accessible pore structure in each precursor. The dependence of activation outcomes on precursor properties has also been demonstrated for biomass-derived carbons.^34^ The potassium detected by EDX provides a possible additional explanation for the low measured areas: retained inorganic material could obstruct pore entrances and contribute non-porous mass to the sample used for area normalization. However, EDX does not quantify the bulk residue fraction, and the present data do not establish how much of the BET-area reduction arises from pore blockage or inorganic mass. These explanations therefore remain possible contributors rather than measured mechanisms.

A BET area is a single measure of nitrogen-accessible apparent surface area, not a pore-size distribution or a micropore-volume measurement. IUPAC guidance notes that nitrogen adsorption at 77 K can be kinetically restricted in very narrow micropores, whereas CO_2_ at 273 K can access some of these pores more readily.^35^ This probe-dependent accessibility means that a modest nitrogen BET area need not imply equally low CO_2_ uptake. In narrow carbon pores, overlapping adsorption potentials from opposing pore walls can favour CO_2_ filling; the proportion of such pores can matter more than total area. Biomass-derived carbons with higher BET areas have accordingly shown lower low-pressure CO_2_ uptake than carbons with narrower pores.^34^ These principles provide a technically plausible explanation for the present trends, but the pore widths and micropore volumes of AS-180, AS-200, and AS-220 cannot be assigned from the reported BET areas, SEM images, or the available CO_2_ uptake points. No pore-size distribution, micropore volume, or total pore volume is inferred from those summary measurements.

Mesopores and macropores may also influence CO_2_ uptake by providing access to internal adsorption sites and affecting gas transport. Kua reported associations between CO_2_ capture and mesopore/macropore volume and porosity in hierarchical biochars, suggesting a role beyond transport alone.^36^ In fixed-bed experiments with biomass-derived activated carbon, Borisut *et al.* likewise found that the micropore-to-mesopore volume ratio affected CO_2_ breakthrough performance.^37^ The present SEM images show surface voids and cracks, but neither SEM nor the reported BET areas establish their size distribution or connectivity. Their contribution to the uptake differences among AS-180, AS-200, and AS-220 therefore remains a plausible explanation, not a measured result.

### X-ray diffraction (XRD) analysis

Fig. 6 presents the baseline-corrected, smoothed, and normalized XRD profiles of the activated hydrochars. The reflection near 2*θ* = 30.05° is treated as an unassigned crystalline reflection, not as a measure of carbon ordering. Disordered carbon is generally associated with broad (002) and (100)/(101) features, whereas discrete sharp reflections can originate from crystalline inorganic residues.^28^ KOH activation and the post-washing EDX detection of K make potassium-containing residues plausible contributors, but elemental detection does not identify a crystalline phase. The normalized profiles cannot establish differences in absolute diffracted intensity. Peak height and width can also depend on phase abundance, preferred orientation, overlapping reflections, and profile processing; without isolating a carbon-associated reflection and accounting for instrumental broadening, they do not establish carbon crystallinity or carbon crystallite growth.

**Fig. 6:**
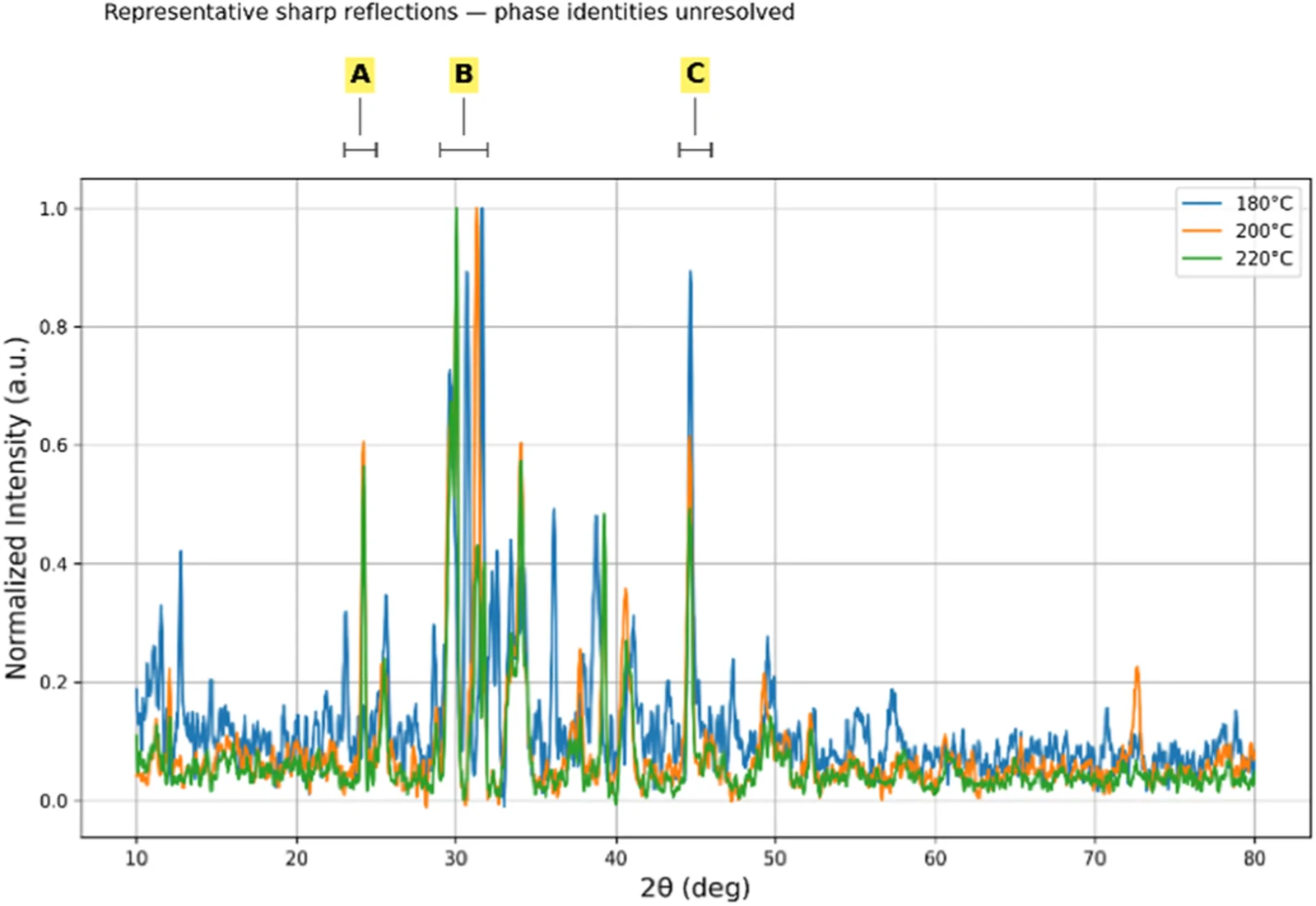
Baseline-corrected, smoothed, and normalized XRD patterns of AS-180, AS-200, and AS-220. Markers A–C indicate representative sharp-reflection regions around 24°, 30–32°, and 45°, respectively; they are not phase assignments. The reported 30.05° reflection remains unassigned. Sample labels denote precursor HTC temperatures; all samples were activated at 700 °C.

Kalicinite and potassium hydrogen carbonate refer to the same compound, KHCO_3_, and must be treated as a single candidate phase. The synthetic kalicinite reference pattern (ICDD 12-292), reproduced in the Handbook of Mineralogy, contains prominent reflections at *d* = 3.67, 2.630, 2.863, 2.816, and 2.97 Å.^38^ Identification therefore requires agreement across several reflections using the experimental X-ray wavelength, rather than agreement with the 30.05° peak alone. Etzi *et al.* reported overlapping reflections from possible potassium carbonate, hydrogen carbonate, and mixed carbonate phases in KOH-activated carbons, illustrating why a single peak is not diagnostic.^28^ A dominant KHCO_3_ phase or a specific phase transformation is consequently not assigned here. The sample labels denote HTC temperatures; all three hydrochars subsequently underwent activation at 700 °C, so the patterns cannot be interpreted as a series of different activation temperatures.

Fig. 6 provides a qualitative diffraction fingerprint of the washed, activated products. Markers A–C highlight representative sharp-reflection regions around 24°, 30–32°, and 45°; these features indicate crystalline constituents even without quantitative phase analysis. Similar reflection regions occur across the samples, but positional coincidence alone does not establish a common phase identity. Phase abundance requires calibrated phase-specific intensities or validated whole-pattern analysis accounting for overlap, absorption, and preferred orientation.^39^ Normalized peak heights therefore cannot supply phase weight fractions. Post-washing EDX detection of K makes retained inorganic residues plausible contributors, without identifying their phases or amounts. XRD consequently adds evidence for crystalline constituents that must be considered alongside the carbon matrix; it does not establish that AS-200's higher CO_2_ uptake arises from greater carbon crystallinity, nor quantify the residue contribution to pore restriction or mass dilution.

### CO_2_ capture

The CO_2_ adsorption capacity of the activated hydrochar samples was systematically evaluated at two pressures (1 bar and 2 bar) and two temperatures (0 °C and 25 °C) to investigate the effects of operating conditions and material structure on capture performance. The results are presented in Table 1, showing distinct trends associated with pressure, temperature, and surface area.

**Table 1:** CO_2_ uptake at absolute pressures of 1 and 2 bar and the indicated measurement temperatures

Sample	Absolute pressure (bar)	Temp. (°C)	BET area (m^2^ g^−1^)	CO_2_ uptake (mmol g^−1^)
AS-180	1	0	37.3	0.37
AS-180	1	25	37.3	0.18
AS-180	2	0	37.3	0.62
AS-180	2	25	37.3	0.38
AS-200	1	0	144.77	1.99
AS-200	1	25	144.77	1.84
AS-200	2	0	144.77	2.5
AS-200	2	25	144.77	1.92
AS-220	1	0	135.2	0.61
AS-220	1	25	135.2	0.39
AS-220	2	0	135.2	0.95
AS-220	2	25	135.2	0.66

For all three samples, the CO_2_ uptake increased with pressure from 1 bar to 2 bar, irrespective of temperature. This observation confirms that higher gas density at increased pressure facilitates enhanced adsorption due to a greater concentration of CO_2_ molecules interacting with the available surface sites. The pressure effect was particularly pronounced in AS-200, where uptake at 2 bar and 0 °C reached 2.5 mmol g^−1^, approximately 25.6% higher than the 1 bar condition at the same temperature. Lower temperatures consistently resulted in higher CO_2_ adsorption across all samples, highlighting the exothermic nature of the adsorption process. The adsorption at 0 °C surpassed that at 25 °C by a notable margin, especially at higher pressures. For instance, AS-220 exhibited a 44% higher uptake at 0 °C (0.95 mmol g^−1^) than at 25 °C (0.66 mmol g^−1^) under 2 bar pressure. This behavior underscores that lower thermal energy reduces molecular agitation and enhances binding between CO_2_ and the adsorbent surface.

KOH activation provides a chemical basis for the material changes. Fischer *et al.* discuss the representative overall reaction 6KOH + 2C ⇌ 2K + 3H_2_ + 2K_2_CO_3_, while noting that actual activation involves precursor-dependent parallel and consecutive reactions.^40^ Carbon consumption and gasification provide routes to pore development, while the fate of potassium-containing products links activation to the subsequent washing step. This provides literature context for the activation treatment; gas evolution, metallic-potassium formation, and individual reaction extents were not measured in the present samples. The products were subsequently cooled and acid/water washed, so the potassium detected afterwards need not retain the chemical form present during activation. The washing reactions discussed above and post-washing EDX/XRD observations therefore concern the final adsorbent, whereas the reaction scheme describes possible chemistry during its preparation. Neither the scheme nor elemental K detection assigns the retained species as K_2_CO_3_ or KHCO_3_.

AS-200 has a BET area only 7.1% higher than AS-220 (144.77 *versus* 135.2 m^2^ g^−1^), but its CO_2_ uptake at 25 °C and 1 bar is 4.72 times higher (1.84 *versus* 0.39 mmol g^−1^). This disparity shows that nitrogen-accessible area alone does not explain performance. Different proportions of CO_2_-accessible narrow pores could produce similar BET areas but different uptake because adsorption potentials overlap more strongly in narrow pores. Nitrogen at 77 K may also be kinetically restricted in pores accessible to CO_2_ at higher measurement temperatures.^34,41^ These mechanisms are plausible, but the present measurements do not establish a pore-size distribution or micropore-volume ranking. Surface chemistry provides a second contribution: the FTIR bands consistent with O–H and C–O functionalities indicate oxygen-containing groups that can modify local polarity and CO_2_ interaction energies. Their effect depends on group type, accessibility, and pore environment; published experimental/simulation work demonstrates that functional groups and pore width influence adsorption affinity and capacity differently.^42^ FTIR intensities do not quantify accessible adsorption sites or establish that AS-200 has the highest surface basicity. Potassium can have competing effects. Carbon-associated K species may strengthen electrostatic interactions with CO_2_, whereas segregated inorganic residues can obstruct pore access or dilute uptake per gram of sorbent.^28^ AS-180 has the highest local EDX K estimate (38.27 wt%) but the lowest uptake at 25 °C and 1 bar (0.18 mmol g^−1^); AS-220 has less local K than AS-200 (16.34 *versus* 18.94 wt%) but also lower uptake. Thus, the local K percentages do not track adsorption capacity monotonically. EDX and XRD do not establish which retained K species are present or accessible. AS-200's higher uptake is therefore consistent with a favourable combination of pore accessibility and surface interactions, without assigning a quantitative contribution to either or inferring micropore collapse from SEM.

At 25 °C and 1 bar, AS-200 adsorbs 1.84 mmol g^−1^, below the reported 3.80 mmol g^−1^ for banana peel activated hydrochar and 3.045 mmol g^−1^ for orange peel activated hydrochar.^11,12^ Thus, AS-200's uptake is approximately 52% and 40% lower than those two activated hydrochars, respectively, under the reported 25 °C and 1 bar conditions. Its performance is moderate among the selected materials. Li *et al.* provide a broader review of strategies used to increase CO_2_ uptake in engineered biochar.^43^ At 0 °C and 1 bar, AS-200 adsorbs 1.99 mmol g^−1^, above the reported 1.01 and 0.63 mmol g^−1^ for the selected orange peel and bamboo biochars, respectively.^19^ These comparisons use the corresponding entries in Table 1 and matching temperature/pressure groups in Table 2. AS-200's maximum of 2.50 mmol g^−1^ was obtained at 0 °C and 2 bar; it is retained as a result for the present samples and is not ranked against literature values measured at 1 bar. Higher pressure increases CO_2_ chemical potential, while lower temperature generally favours exothermic adsorption, so comparing maxima measured under different conditions would confound material properties with operating conditions. Even after matching temperature and nominal pressure, gas composition, sample pretreatment, measurement method, and uptake definition can influence cross-study comparisons. The numerical comparisons therefore describe the selected reported cases rather than establish universal adsorbent superiority. They also do not isolate the contributions of pore structure and surface chemistry or establish performance under direct air capture conditions. The selected studies in Table 2 show that CO_2_ uptake varies substantially among biochars and activated hydrochars; there is no single representative capacity for either material class. For AS-200, 1.84 mmol g^−1^ at 25 °C and 1 bar is equivalent to approximately 0.081 g CO_2_ per g sorbent, while its maximum of 2.50 mmol g^−1^ at 0 °C and 2 bar is approximately 0.110 g g^−1^. These are pure-CO_2_ adsorption loadings for KOH-activated hydrochar, not the amount recoverable in each industrial adsorption–regeneration cycle. As discussed in the next section, assessing industrial use requires measurements at relevant CO_2_ partial pressures, including humid mixed-gas breakthrough and cyclic working capacity.

**Table 2:** Condition-matched CO_2_ uptake of AS-200 and selected biomass-derived adsorbents at 1 bar^a^

Adsorbent	Temp. (°C)	*P* (bar)	CO_2_ uptake (mmol g^−1^)	Ref.
Banana peel activated hydrochar (BP-180)	25	1	3.80	Goel *et al.* 2021 (ref. 5)
Orange peel activated hydrochar (OP-220)	25	1	3.045	Goel *et al.* 2021 (ref. 5)
AS-200	25	1	1.84	This work
Orange peel biochar	0	1	1.01	Kwon *et al.* 2025 (ref. 19)
Bamboo biochar	0	1	0.63	Kwon *et al.* 2025 (ref. 19)
AS-200	0	1	1.99	This work

a The 0 °C literature measurements in ref. 19 were reported at 273 K. Uptake values follow the original papers, including the mmol g^−1^ values in Table 2 of ref. 19.

### Implications for industrial CO_2_ capture

Industrial capture depends on CO_2_ partial pressure and dynamic bed utilisation. For illustration, a gas containing 15 vol% CO_2_ at a total pressure of 1 bar absolute has a CO_2_ partial pressure of 0.15 bar; the present pure-CO_2_ measurements at 1–2 bar do not quantify uptake at that condition. Arjona-Jaime *et al.* reported 2.42 mmol g^−1^ for Ca-modified biochar at 25 °C and 1 bar total pressure in a gas containing 15% CO_2_. Because the material and gas composition differ, this value cannot be ranked directly against our pure-CO_2_ uptake.^44^ Manyà *et al.* examined physically activated vine-shoot and wheat-straw biochars in cyclic packed-bed tests with dry and humid gases, and found that water vapour shortened breakthrough times.^9^ Water adsorption at hydrophilic sites followed by clustering within pores provides a mechanism for such interference, with its extent depending on surface chemistry and pore structure.^31^ The FTIR O–H/C–O assignments in the present activated hydrochars identify potentially water-affine environments, but they do not measure wettability. The pure-CO_2_ uptake measurements do not establish hydrophobicity or humid mixed-gas performance. This illustrates why gas composition and contact time matter for usable bed capacity. In cyclic operation, working capacity is the difference between the loadings reached at adsorption and desorption conditions. It therefore depends on regeneration as well as adsorption. Heat released during adsorption can raise the local bed temperature and reduce uptake, while finite diffusion and cycle times can prevent full utilisation of equilibrium capacity. Also, future studies may integrate statistical and machine learning approaches to optimize hydrothermal carbonization and activation parameters for simultaneous improvement of adsorption performance and environmental sustainability.^45^

Sorbent form introduces further trade-offs during scale-up. Smaller particles shorten intraparticle diffusion paths but increase packed-bed pressure drop. Pelletisation improves handling and limits entrainment, while binders may obstruct pore access and larger particles can slow mass transfer. Mammarella *et al.* studied activated vineyard-pruning biochar in laboratory pressure-swing adsorption at 5, 7, and 9 bar absolute, and identified shaped sorbents and transport/temperature effects as considerations for larger beds.^46^ Accordingly, selection for an industrial bed involves volumetric working capacity, breakthrough behaviour, mechanical integrity, and regeneration energy alongside uptake per gram. Volumetric working capacity also depends on packed bulk density, so uptake per gram alone cannot determine the required bed volume. For areca sheath, the KOH requirement, activation at 700 °C, and washing also carry material and energy burdens. The present LCA identifies upstream burdens under its stated inventory; it does not establish industrial throughput, capture purity, or cyclic sorbent lifetime.

### Life cycle assessment

Fig. 7a and b compares the baseline GWP_100_ results on separate production and CO_2_-processing bases. Production of 1 kg of activated hydrochar gives 941, 235, and 611 kg CO_2_-eq. for AS-180, AS-200, and AS-220, respectively. AS-200 is rounded consistently to 235 from the original Sankey total of 2.347 × 10^2^ kg CO_2_-eq. These are deterministic inventory results, not statistical means. The production inventory assigns the reported burden to upstream chemical and energy supply; zero direct foreground emissions in that model do not establish that the physical synthesis releases no emissions. Reducing KOH demand or reactor energy use can reduce these upstream burdens, even when the emissions occur outside the synthesis facility.

**Fig. 7:**
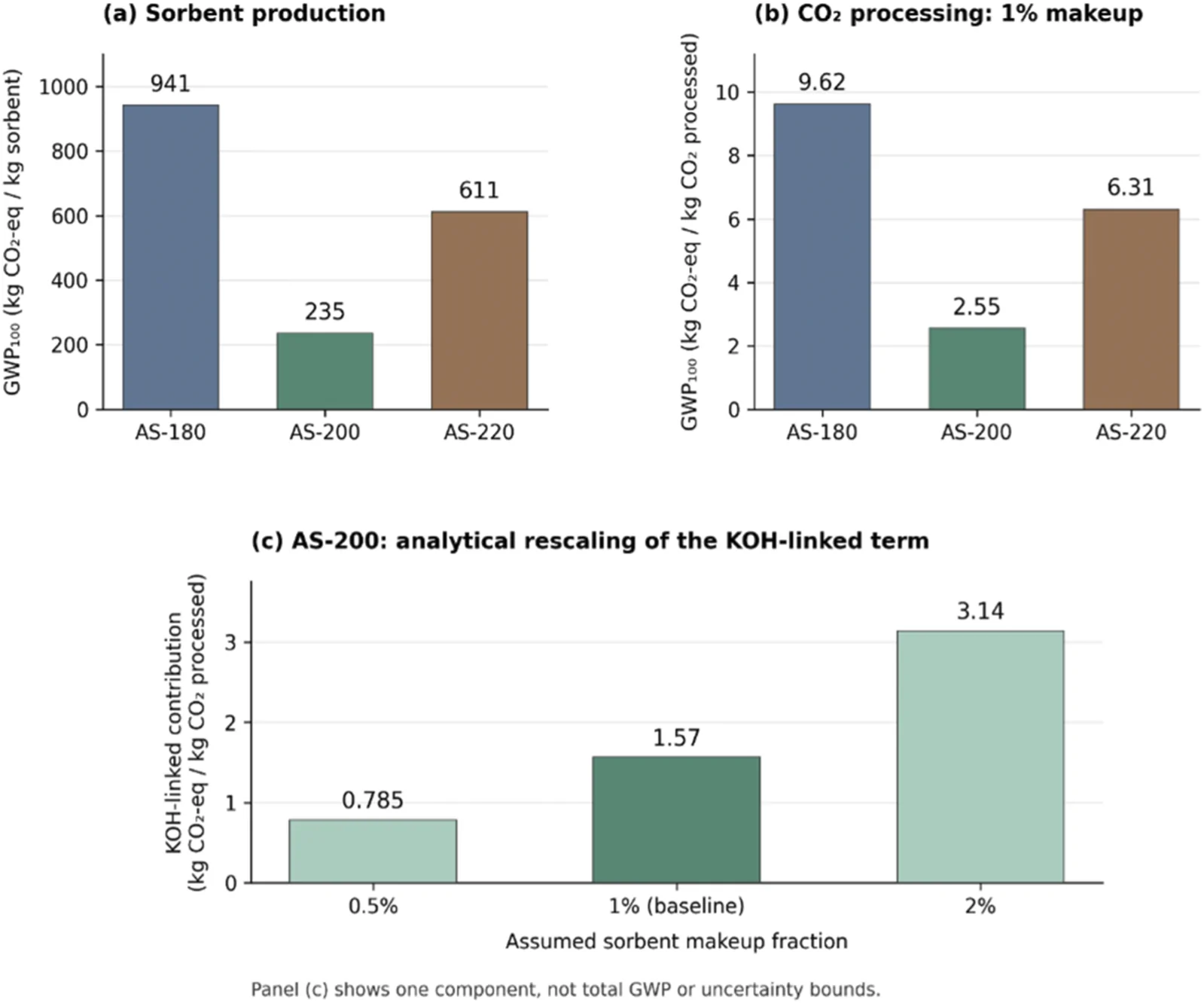
Baseline GWP_100_ for (a) production of 1 kg of activated hydrochar and (b) processing of 1 kg of CO_2_ with assumed 1% sorbent makeup. (c) Analytical rescaling of the AS-200 KOH-linked makeup contribution at illustrative fractions of 0.5%, 1%, and 2%, with other inventory conditions fixed.

The original contribution analysis identifies similar major pathways across the three samples: KOH production through chlor-alkali electrolysis contributes about 66–68% of total production GWP_100_, industrial gas supply about 16%, and hydrochloric acid manufacture about 9–10%. The ranges describe contribution shares across the modelled cases; they are not uncertainty intervals.

Table 3 summarizes upstream pathways and their drivers. In the AS-200 Sankey output, medium-voltage electricity, high-voltage electricity, voltage transformation, and fossil electricity-generation nodes have cumulative shares of approximately 21.4%, 22.4%, 28.4%, and 11.23% of the total, respectively. These nodes occur within connected supply chains, so their burdens overlap and cannot be added as independent contributions or stacked with the full KOH-production burden. Their purpose is to trace electricity-related hotspots within the dominant KOH pathway. The attribution identifies electricity supply as an important driver; it does not establish that reaction conditions or chemical demand are environmentally irrelevant.

**Table 3:** Upstream pathways and their drivers. Percentages refer to total production GWP_100_; nested electricity and salt nodes are cumulative and overlap their parent pathways, so the rows must not be summed as independent contributions

Process	Dominant upstream driver	Energy carrier	Share of total GWP_100_ (%)
KOH production (chlor-alkali electrolysis)	Electricity-intensive operations	Fossil electricity	66–68
Electricity supply chain	Generation + transformation losses	Lignite/coal mix	20–25 (nested electricity nodes)
Sodium chloride production	Mineral processing	Fossil energy	16–23 (nested salt nodes)
Industrial gas supply	Fuel extraction & processing	Natural gas	16
Hydrochloric acid production	Energy + chemical synthesis	Fossil-based	10

It is also seen that sodium chloride production emerges as a significant upstream contributor within the chlor-alkali system, reflecting the energy intensity associated with mineral extraction and processing. In addition, hydrochloric acid production contributes approximately 10% of total GWP_100_, indicating that post-activation washing and purification steps impose a non-negligible environmental penalty. These findings show that auxiliary chemicals and material precursors contribute significantly to the overall footprint and should not be treated as environmentally neutral inputs.

Industrial gas supply contributes approximately 16% of production GWP_100_ across the modelled samples. Thermal energy demand also appears in nested heat and steam supply pathways, including natural-gas-based heat. These cumulative nodes should not be summed again with their parent pathways. The contribution analysis therefore supports attention to thermal demand and fuel supply, while the baseline bar charts provide a direct comparison of total impacts.

AS-180 exhibits the highest GWP_100_ due to a significant upstream electricity and thermal energy demand per unit of activated hydrochar. AS-220, on the other hand, shows partial mitigation but remains constrained by reduced material yields at higher HTC severity. In contrast, AS-200 achieves the lowest climate impact by minimizing upstream energy and material demand without altering the underlying process structure. AS-200 therefore has the lowest impact within the included production inventory; its ranking after wastewater treatment depends on the sample-specific additional burdens.

Under the baseline inventory with 1% sorbent makeup, the adsorption–regeneration stage gives GWP_100_ values of 9.62, 2.55, and 6.31 kg CO_2_-eq. per kg CO_2_ processed for AS-180, AS-200, and AS-220, respectively. AS-200 has the lowest modelled impact under these common assumptions. Its KOH-linked contribution associated with fresh-sorbent makeup is approximately 1.57 kg CO_2_-eq. per kg CO_2_ processed, or about 62% of the 2.55 kg total. Replacement carries upstream burdens from manufacturing new activated hydrochar, including KOH production, so an assumed replacement input can materially affect the capture-stage result even when adsorption uptake is favourable. Other sorbent-production contributions may also accompany replacement; the KOH-linked value is not the whole makeup burden. The baseline result exceeds the benchmark of 1 kg CO_2_-eq. per kg CO_2_ processed, but this conclusion applies to the selected inventory and does not establish the environmental performance of a material with a different replacement requirement. Production-stage impacts per kilogram of hydrochar remain separate from this assumed rate of use-stage replacement.

The influence of makeup can be explained through the inventory balance. If the CO_2_ processing basis, adsorption performance, regeneration energy, and unit sorbent-production burden are held fixed, the dependence is *G*(*r*) = *A* + (*r*/*r*_0_)*B*, where *r* is the makeup fraction, *r*_0_ = 0.01 is the baseline fraction, *B* is the replacement-related contribution at that baseline, and *A* contains contributions held unchanged. *G*(*r*), *A*, and *B* are expressed as kg CO_2_-eq. per kg CO_2_ processed. Thus, only the replacement-related component scales directly with makeup; the total GWP need not vary in the same proportion. Reduced makeup also leaves energy-related and other process burdens in place. This relation describes the inventory dependence and does not model capacity degradation. More cycles increase cumulative inputs and cumulative CO_2_ processing; with unchanged performance and replacement fraction, they do not necessarily increase GWP per kilogram processed. Capacity loss could instead raise the sorbent and energy demand per kilogram of CO_2_, but uptake at a single adsorption condition does not determine cyclic working capacity, which depends on adsorption and desorption loadings. Accordingly, the present results do not establish sorbent lifetime, a verified replacement rate, or uncertainty bounds for the capture-stage GWP. The environmental conclusions are restricted to the baseline scenario rather than extrapolated to unmeasured cyclic operation.

Fig. 7c gives a limited analytical sensitivity illustration for the known AS-200 KOH-linked makeup term: *C*_k_(*r*) = 1.57(*r*/0.01) kg CO_2_-eq. per kg CO_2_ processed. At illustrative makeup fractions of 0.5%, 1%, and 2%, this term is 0.785, 1.57, and 3.14 kg CO_2_-eq., respectively. Thus, halving or doubling makeup changes this contribution by −0.785 or +1.57 relative to the baseline. The rescaling holds adsorption performance, regeneration energy, and unit KOH supply burden fixed. *C*_k_ is only part of B in the preceding inventory balance; these values are not total use-stage GWP. The separately reported KOH term cannot establish the response of all replacement-related inputs or a sample ranking at different makeup rates. For AS-200, a 25% reduction in the baseline CO_2_-processing GWP_100_ would require a decrease of about 0.64 kg CO_2_-eq. per kg CO_2_ processed, from 2.55 to 1.91. The identified KOH-linked fresh-sorbent contribution is 1.57 kg CO_2_-eq.; reducing this contribution by approximately 41% would meet that target if all other contributions and adsorption performance remained fixed. Future work should therefore test lower KOH-to-hydrochar ratios than the present 3 : 1 ratio, measure sorbent lifetime to replace the assumed 1% makeup rate, and investigate potassium recovery.^47^ Each option must be assessed against activated-hydrochar yield, cyclic CO_2_ working capacity, and any added recovery or wastewater-treatment inputs. This calculation sets a research target; it is not a demonstrated 25% reduction or a revised LCA result.

Parameter sensitivity and data uncertainty answer different questions. Fig. 7c illustrates the consequence of changing one assumed input; it does not estimate a probability distribution. Error bars on total GWP require quantified uncertainty in inventory inputs and its propagation through the model. The reported results do not provide such intervals, so error bars are omitted rather than inferred from differences between HTC samples or from illustrative scenario ranges. The comparison remains conditional on the baseline inventory and its exclusions.

### Screening estimate for washing-effluent treatment

Acid washing converts accessible alkaline potassium species into soluble salts; neutralisation controls pH but does not remove dissolved potassium or chloride. On a loading basis, 3 kg KOH per kilogram of dried HTC hydrochar corresponds to 3 × (74.55/56.11) = 3.99 kg KCl-equivalent if all added potassium enters the wash liquor and sufficient HCl is available. This is a stoichiometric loading-equivalent, not a measured salt discharge: potassium retention, thermal losses, native minerals, and other effluent constituents are not quantified. It also refers to precursor hydrochar, not the final activated-product functional unit.

For an electricity-only screening estimate, Δ*G* = (*W*/1000) × *e* × *F*, where *W* is washing-effluent volume in L kg^−1^ of final activated hydrochar, *e* is treatment electricity in kWh m^−3^ of feed, and *F* is the electricity climate factor in kg CO_2_-eq. per kWh. Wang *et al.* reported modelled low-salt-rejection reverse-osmosis requirements of 2.4–8.0 kWh m^−3^ and a mechanical-vapour-compression comparator of 20–25 kWh m^−3^ for concentrating 0.1 to 1.0 M NaCl.^48^Table 5 scales these literature benchmarks to each 100 L of effluent and illustrates GWP using *F* = 0.5 kg CO_2_-eq. per kWh. The 100 L normalization and *F* value are illustrative parameters, not measurements or inferred inventory values for the present study.

**Table 4:** Tentative assignments of the FTIR band regions labelled in Fig. 4

Band	Region (cm^−1^)	Tentative assignment	Interpretation and references
A	3100–3500	Hydrogen-bonded O–H stretching	Hydroxyl-containing groups and/or adsorbed water; not proof of intact biomass polymers^29,30^
B	Around 2350	CO_2_ asymmetric stretching	Compatible with residual atmospheric/background CO_2_; not assigned to C≡C or bound CO_2_ (ref. 30 and 32)
C	1600–1750	C=O-containing envelope	Conjugated carbonyl and adsorbed-water bending (∼1640 cm^−1^) may overlap; individual species unresolved^29^
D	1500–1600	Aromatic skeletal C=C vibrations	Possible conjugated C=O overlap; does not establish graphitic ordering^29^
E	1350–1450	C–H deformation and possible carbonate absorption	Organic/inorganic overlap near 1400 cm^−1^; retained K supports plausibility, not carbonate-phase identification.^29,31^
F	1000–1200	C–O/C–O–C vibrations	Overlapping oxygen-containing environments, including alcohols, phenols, and ethers^29^

**Table 5:** Normalized electricity-only screening estimate for washing-effluent treatment

Literature benchmark (ref. 48)	Electricity (kWh m^−3^ feed)	Electricity per 100 L (kWh)	Illustrative GWP per 100 L (kg CO_2_-eq.)
Four-stage low-salt-rejection reverse osmosis	2.4–8.0	0.24–0.80	0.12–0.40
Mechanical vapour compression comparator	20–25	2.00–2.50	1.00–1.25

The corresponding electricity-only increments are 0.12–0.40 and 1.00–1.25 kg CO_2_-eq. per 100 L. For *W* L kg^−1^ of activated hydrochar, multiply these normalized increments by *W*/100; for the CO_2_-processing functional unit, also multiply by the inventory mass of fresh sorbent supplied per kilogram of CO_2_ processed. These values illustrate scaling with wash volume and treatment route. The NaCl benchmarks are proxies, not validated designs or upper/lower bounds for the potassium-rich washing effluent.

A complete treatment inventory would additionally include any residual pH adjustment, solids/organic removal, membrane or evaporator consumables, and concentrate/sludge disposal. HCl and water manufacture already included for sorbent washing should not be counted again; only additional treatment inputs belong in the omitted burden. No recovery credit is assigned. Neutralisation alone is not desalination, and concentrating brine is not complete disposal. Salinity-related ecological effects are also outside the reported climate-change indicator. Consequently, Table 5 supplies a conditional screening estimate without establishing a negligible treatment burden or a treatment-inclusive ranking of the three sorbents.

## Conclusions

The present study examines the preparation of hydrochar from areca sheath *via* hydrothermal carbonization. HTC was conducted at temperatures of 180 °C, 200 °C, and 220 °C, followed by chemical activation using potassium hydroxide (KOH) to improve porosity and surface area. Characterization analyses such as XRD, FTIR, SEM, and EDX were essential in identifying the structure and composition of the hydrochar. XRD showed crystalline reflections potentially associated with retained inorganic residues; these reflections do not establish increasing carbon-matrix crystallinity with HTC temperature. FTIR bands were consistent with O–H, C–O, and overlapping carbonyl/aromatic vibrations, with a possible carbonate contribution near 1400 cm^−1^. These qualitative assignments do not quantify functional-group loss or establish graphitic ordering. Morphological features were examined using SEM images; AS-180 showed initial pore formation, AS-200 displayed widespread surface cracking and visible pore development, while AS-220 exhibited structural collapse, likely due to excessive thermal exposure. BET surface area analysis supported these findings, with AS-200 showing the highest specific surface area of 144.77 m^2^ g^−1^. The local EDX carbon estimates were 21.98 wt% for AS-180 and 54.68 wt% for AS-220; these values represent the analysed regions rather than bulk carbon purity. CO_2_ capture tests indicated that AS-200 had the best adsorption capacity, reaching 2.5 mmol g^−1^. AS-200 and AS-220 differ by only 7.1% in BET area but by 4.72-fold in CO_2_ uptake at 25 °C and 1 bar. Together with the oxygen-containing FTIR environments and post-washing potassium, this comparison shows why nitrogen-accessible area and local elemental percentages are insufficient descriptors of adsorption. The chemical interpretation considers surface interactions, pore accessibility, and inorganic-residue retention jointly, without assigning a measured causal contribution to each.

In addition to experimental performance, life cycle assessment quantified the climate change impacts associated with sorbent production and use. On a production basis, AS-200 exhibited the lowest GWP_100_ (235 kg CO_2_-eq. per kg activated hydrochar), compared to AS-220 (611 kg CO_2_-eq. per kg) and AS-180 (941 kg CO_2_-eq. per kg), with chlor-alkali electrolysis for KOH production contributing to about 67% of total emissions. With an assumed 1% fresh-sorbent makeup in the baseline adsorption–regeneration inventory, GWP_100_ values were 2.55, 6.31, and 9.62 kg CO_2_-eq. per kg CO_2_ processed for AS-200, AS-220, and AS-180, respectively. These values show that adsorption uptake alone is insufficient to establish climate benefit under the selected inventory. AS-200 has the lowest capture-stage impact in this scenario, but the finding remains conditional on the assumed replacement input and regeneration inventory. Cyclic working capacity, capacity retention, and the actual sorbent replacement requirement were not established, so these results cannot demonstrate long-term operating performance. The baseline assessment identifies upstream sorbent production and energy supply as priorities for reducing the modelled climate burden. The baseline GWP values exclude wastewater treatment. The normalized screening estimate identifies an additional burden dependent on effluent volume, treatment route, and electricity supply.

## Author contributions

SM conceptualized the work, prepared the visualizations, conducted data analysis, and drafted the original manuscript; RKG and JP assisted in visualization, analysis, and editing of the original manuscript; SS was involved in data analysis and reviewed the original manuscript; PD supervised, investigated, and reviewed the original manuscript.

## Conflicts of interest

There are no conflicts to declare.

## Data Availability

All processed data supporting the findings of this study—including structural and morphological characterizations, CO_2_ adsorption measurements, and key Life Cycle Assessment (LCA) results are presented within the article. Due to their large file sizes, the raw instrument datasets and the complete openLCA computational models are available from the corresponding author upon reasonable request.
